# How to Initiate Fecal Microbiota Transplantation in Developing Countries Using the Behavior Economics Concept of “Choice Architecture”

**DOI:** 10.3389/fmed.2021.746230

**Published:** 2022-01-12

**Authors:** Chatuthanai Savigamin, Nuttida Mahakit, Suchinda Stithit, Chatpol Samuthpongtorn

**Affiliations:** ^1^Department of Parasitology, Faculty of Medicine, Chulalongkorn University, Bangkok, Thailand; ^2^Faculty of Medicine, Chulalongkorn University, Bangkok, Thailand; ^3^Century Pharmaceuticals, Inc., Indianapolis, IN, United States

**Keywords:** fecal microbiota transplantation, gastrointestinal diseases, stool bank, behavior economics, choice architecture, nudges

## Introduction

Fecal Microbiota Transplantation (FMT) is a transfer of stool from a “healthy” donor to restore eubiosis (healthy microbiome) in a recipient believed to harbor an altered colonic microbiome (dysbiosis) resulting in disease development (1, 2). FMT is also referred to as stool transplantation, fecal transplantation, fecal flora reconstitution, or fecal bacteriotherapy. Though it is mostly employed in the treatment of *Clostridioides difficile* (formerly known as *Clostridium difficle*), FMT is now being investigated for its mechanism in the treatment of inflammatory bowel disease, irritable bowel syndrome, hepatic encephalopathy, and other conditions (2). Recently FMT has gained popularity in both the public media as well as peer-reviewed literature (1). However, one of the challenges obstructing a successful FMT program is a negative perception regarding FMT from both physicians and public citizens.

Nowadays, only *Clostridioides difficile* is approved for treatment using FMT. Fecal microbiota transplantation has the potential to cure *Clostridioides difficile* through a direct pathway (short-chain fatty acids and bile acids), nutrition competition, and bacteriophages that are unrelated to the host and are transferred directly following fecal microbiota transplantation. As a result, *Clostridioides difficile* can be cured through the use of a healthy donor who is not required to be specific to each patient (**Donor independent**) (2). Gastroenterologists can choose any healthy individual to donate stool. It is not necessary to have a program for donor recruitment for finding a perfect stool donor. However, many other diseases, such as irritable bowel syndrome, inflammatory bowel disease, and hepatic encephalopathy, require a specific, perfect donor. This is because FMT contributes to the development and treatment of these disorders in an indirect pathway (through bile acid and short-chain fatty acid metabolism) and is influenced by host-associated factors (**Donor dependent**) (2). Consequently, if we want to develop FMT as a successful novel therapeutic approach, we'll need a donor recruitment program to establish pools of perfect stool donor for research and treatment.

One of the main challenges facing public citizens is that feces is a “Yucks Term.” Feces is associated with a dirty and unpleasant image. It's challenging to convince them that this is a novel treatment. We believe social media platforms should rename “feces” to more attractive words. This is what is referred to as “Punning.” For example, Nerlich and Koteyko (3) redefined FMT as “vitalism,” which is synonymous with probiotic (4). Furthermore, some asserted that FMT was a supernatural power called “God's probiotic” (5). Thus, FMT was linked with the socially acceptable because they are familiar with probiotics in medicine (4).

We applied the Nudge principle to promote a positive perception regarding FMT to both physicians and public citizens. The Nudge principle was invented by Richard H Thaler since the early twentieth century, and he won a Nobel Prize in 2017 for his contribution in Nudge principle. Nudge is a behavior intervention designed to change people's behavior indirectly by changing the environment and situation to allow for a better choice and decision making (6). Nudge is a well-known principle employed in the business, economics, and healthcare domains. This principle is based on incentive, understanding mapping, default choice, feedback, relative comparison, expansion of the important outcome and structural complex choices (6). We believe the Nudge principle could lead to a structure that allows a successful establishment of FMT programs in the developing countries.

## Discussion and Suggestions

We propose four essential steps for initiating fecal microbiota transplantation.

1. Finding a perfect stool donor.
2. Initiating a clinical trial.
3. Establishing a stool standard for use in other research trials.
4. Establishing a clinical center for the transplantation of fecal microbiota.

We focused on the first two steps in forming the fecal microbiota transplantation in this paper. We established nine interventions based on the Nudge principle which will be incorporated into each step of initiation of fecal microbiota transplantation.

1. Finding perfect stool donors—numerous steps should be taken to find perfect stool donors, such as completing a questionnaire to select an appropriate donor, collecting blood samples for further investigation, receiving stool specimens and investigating the stool specimens for proper criteria to be considered as a perfect donor (7). We propose that by incorporating Nudge theories and our suggestion, we can make these steps easier and more successful by nudging the stool donors.

### Intervention 1: Certification

Openbiome (the world's largest non-profit stool bank) (8) as well as Asia Microbiota bank (Asia's largest stool bank) provide financial reward as an incentive to motivate fecal microbiota donors to continue their donation.

Apart from financial incentives, we propose a certificate of merits be given to fecal microbiota donors. This certificate will also indicate that the donor is healthy and passes the health examination screening. Additionally, this certificate can be used for a discount to future healthcare-related cost. This method was count as an incentive in nudge principle.

### Intervention 2: Complimentary Breakfast

Most people defecate in the morning; therefore, fecal microbiota donation in the morning is usually preferred. A complimentary breakfast serves as both an incentive and is part of a pleasant environment prior to the donation. Additionally, it also triggers a gastrocolic reflex stimulating the defecation.

### Intervention 3: Elegant Home-Like Toilet

Defecation can be challenging in an unfamiliar environment. Therefore, arranging a toilet that replicates an elegant home-like environment would be helpful to facilitate ease in defecation of fecal microbiota donors.

### Intervention 4: Provide Essential Information

**Figure 1:**
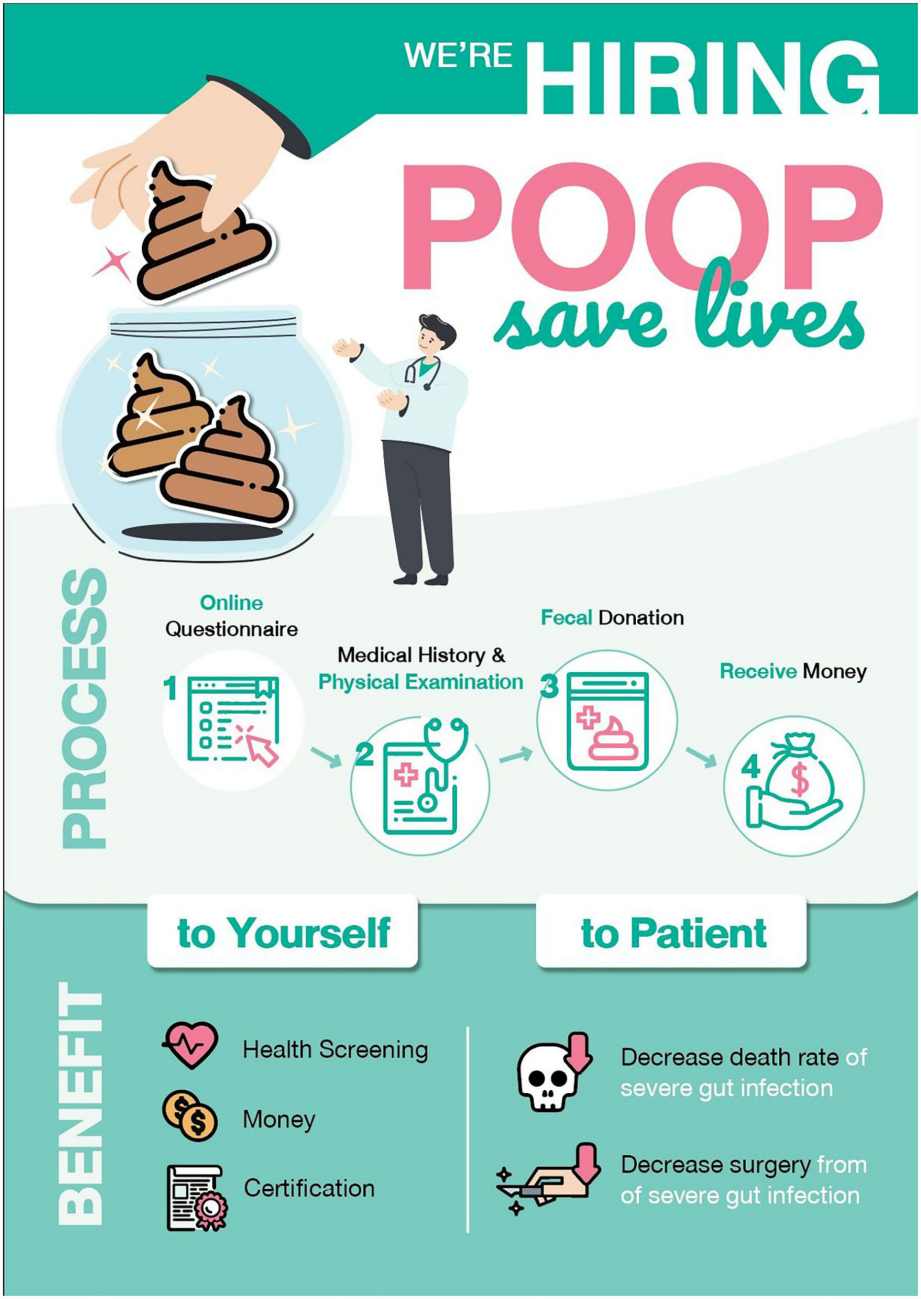
Infographic about stool donation created by our team. It contained not much excessive amounts of data and text, eye-catching picture and an organized story.

Information should contain pros and cons of the fecal microbiota donation along with the procedure in detail. Excessive information and text should be avoided. The infographic should attract attention and should be self-explanatory; the narration should be concise and easy to understand; and the presented data should be distinctive (9). We propose our infographic in Figure 1. By using this method, we incorporated the principle of understand mapping into our suggestion.

### Intervention 5: Advertisement Imaging

If we want to make it easier to find perfect stool donors, one of the most essential aspects is to shift the imaging of fecal microbiota transplantation away from poop transfer and toward more sophisticated imaging such as a natural probiotic cocktail, God's probiotic, etc. This shift in perception should help persuade more stool donors to donate fecal microbiota.

### Intervention 6: Online Community for Fecal Microbiota Transplantation

Social networking has become an essential part in this modern era. It can be a powerful tool to enhance the awareness of FMT and expand the donor pool. Multiple channels such as a Facebook page, an official Line group, or a webpage should be utilized to capture a larger interest group of donors. We had known from previous reports that people tend to conform to other people, and these effects especially increase when the size of other people increase. This online community should be a useful tool in finding perfect donors when everybody is talking about fecal donation. This type of intervention in nudge theories is called feedback (6).

2. Initiating clinical trial: After finding perfect stool donors, we must screen patients for a variety of diseases. Depending on our treatment objective, we can use selected stool from the perfect donor pool. In this section, we want to nudge the gastrointestinal medical doctors or referring physicians to pay attention or use this type of intervention. We suggest the following intervention for those processes.

### Intervention 7: Comparison Between Our Country and Neighboring Countries

We propose using an infographic comparison between various core domains of each FMT program. This information can be utilized to continuously improve the program performance. However, given the difference in the culture and philosophy of each country, certain interventions may not always yield similar results. This intervention would nudge the GI doctors and referring doctors to believe that this FMT program has been widely used in their neighborhood. It will create the comparison which should nudge the doctors to use more FMT and do more research about them.

### Intervention 8: Emphasize the Benefit of Fecal Microbiota Transplantation

We propose emphasizing how FMT could impact people's lives in a positive way and how fecal microbiota donor could have an enormous impact on helping others who suffer. For example, people with severe refractory *Clostridioides difficile* infection have a mortality rate of 43.2%. However, with FMT, the mortality rate is reduced to 12.1%. FMT can substantially decrease the requirement for colectomy in these patients from 31.8 to 7.6% (9, 10). We should simplify the essence of this intervention to nudge the doctors toward the belief that this FMT program is interesting and worth doing research on and worth referring patients to.

Because developing countries often lack resources and may encounter difficulties initiating FMT for only *Clostridioides difficile* infection, we should emphasize the impact and potential of FMT as a therapeutic target for various diseases. For example, FMT could be effective in curing inflammatory bowel disease, irritable bowel syndrome, and hepatic encephalopathy (2). Additionally, several studies have demonstrated that FMT may be beneficial in the treatment of metabolic syndrome, obesity, autism, Parkinson's disease, multidrug-resistant organism infections and autoimmune disorders (11–13). We should emphasize that, while initiating FMT in developing countries was challenging, if we succeed, we will have therapeutic targets for not only gastrointestinal disease but also for significant extra-gastrointestinal diseases that were the serious burden in developing countries.

### Intervention 9: Default Choice of FMT in Comparison Between Developed Countries and Developing Countries

FMT has been included in several expert guidelines of standard practice for the treatment of recurrent and refractory *Clostridioides difficile* infection in developed countries in the recent years (14), and it is the indication approved by the United States (US) Food and Drug Administration (FDA) since 2013 (15). On the other hand, FMT was not included in clinical practice in developing countries, including Thailand (16). This feature obscured the application of FMT in clinical practice. We propose that incorporating FMT into clinical guidelines for *Clostridioides difficile* infection by adapting existing guidelines from developed countries in medical school and hospital training could benefit physicians to start FMT as standard practice. By adapting guidelines from developed countries in establishing FMT as a default choice for general physicians because physicians are more likely to follow the established guidelines.

3. Establishing a stool standard for use in other research trials.

4. Establishing a clinical center for the transplantation of fecal microbiota.

Steps 3 and 4 are the final steps, which require a multidisciplinary team comprised of dedicated medical doctors, scientists, pharmacists, and other associated staffs. It requires many complicated steps. However, if we can build a foundation in the first step, the subsequent steps will be a lot simpler.

We believe these interventions will promote the awareness and enable an establishment of a robust FMT program in Thailand and other developing countries.

## Author Contributions

CSav had the idea of writing the manuscript. CSav, NM, and CSam drafted the manuscript. SS revised the manuscript. CSam corrected and reviewed the manuscript. All authors approved the final version.

## Conflict of Interest

SS is employed by Century Pharmaceuticals, Inc. The remaining authors declare that the research was conducted in the absence of any commercial or financial relationships that could be construed as a potential conflict of interest.

## Publisher's Note

All claims expressed in this article are solely those of the authors and do not necessarily represent those of their affiliated organizations, or those of the publisher, the editors and the reviewers. Any product that may be evaluated in this article, or claim that may be made by its manufacturer, is not guaranteed or endorsed by the publisher.
